# STOP-Bang and the effect on patient outcome and length of hospital stay when patients are not using continuous positive airway pressure

**DOI:** 10.1007/s00540-014-1848-0

**Published:** 2014-05-29

**Authors:** Monika A. Proczko, Pieter S. Stepaniak, Marcel de Quelerij, Floor Haak van der Lely, J. (Frans) Smulders, Lukasz Kaska, Mohammed A. Soliman Hamad

**Affiliations:** 1 Gdańsk Medical University, Gdańsk, Poland; 2 Catharina Hospital Eindhoven, Michelangelolaan 2, 5623 EJ Eindhoven, The Netherlands

**Keywords:** Obstructive sleep apnea syndrome, Morbid obesity, Postoperative complications, Sudden death

## Abstract

**Background:**

In patients undergoing surgical interventions under general anesthesia, obstructive sleep apnea syndrome (OSA) can cause serious perioperative cardiovascular or respiratory complications leading to fatal consequences, even sudden death. In this study we test the hypothesis that morbidly obese patients diagnosed by a polysomnography test and using continuous positive airway pressure (CPAP) therapy have fewer and less severe perioperative complications and a shorter hospital stay than patients who have a medical history that meets at least three STOP-Bang criteria and are not using CPAP therapy.

**Methods:**

Postoperative hospital stay and pulmonary complications were analyzed in three groups of morbidly obese patients undergoing bariatric surgery (Roux-en-Y gastric bypass and laparoscopic sleeve gastrectomy) between January 2009 and November 2013 (*n* = 693). Group A comprised 99 patients who were preoperatively diagnosed with OSA based on polysomnography results. These patients used CPAP therapy before and after surgery. Group B consisted of 182 patients who met at least three STOP-Bang criteria but who were not diagnosed with OSA based on polysomnography results. These patients did not use CPAP. Group C, the reference group, comprised 412 patients who scored one to two items on the STOP-Bang.

**Results:**

During the perioperative period, Group B patients had a significantly (*p* < 0.001) higher cumulative rate of pulmonary complications, worse oxygen saturation, respiratory rates, and increased length of stay in hospital. There was also two cases of sudden death in this group.

**Conclusion:**

Based on these results, we conclude that patients meeting at least three STOP-BANG criteria have higher postoperative complications and an increased length of hospital stay than patients using CPAP.

## Introduction

One of the most common sleep disorders is sleep apnea, and it is believed that approximately 1–10 % of adults are affected by obstructive sleep apnea syndrome (OSA). OSA is characterized by decreased rates of breathing or an absence of breathing during sleep [1] and is a well-known risk factor associated with morbidly obese individuals [2]. In obese patients, OSA is attributed to a reduction in pharyngeal cross-sectional area due to peripharyngeal fat deposition [3]. In patients undergoing bariatric surgery, OSA is a risk factor for postoperative complications, such as cardiac arrhythmias and decompensation, pulmonary embolism and sudden death [4–8]. The exact mechanisms underlying these associations are complex and not yet fully understood [9]. The cardio-respiratory consequences of OSA may exacerbate in the postoperative period due to the adverse effect of general anesthetics and pain treatment for breathing control and upper airways muscle tone. Based on such information, many authors have suggested that it is important to identify patients suffering from OSA [11, 12]. Earlier studies of preoperative sleep disorders prior to bariatric surgery demonstrated an overall prevalence rate of OSA ranging from 48 to 91 % [13, 14]. Not all morbidly obese patients are routinely tested and diagnosed for OSA by a polysomnography (PSG) test, which is a test that enables OSA to be diagnosed objectively [15, 16]. Patients diagnosed for OSA receive the advice to use continuous positive airway pressure (CPAP), which is effective in treating obstructive sleep apnea [17, 18]. Clinicians often use the STOP-Bang questionnaire to estimate the OSA risk involved [19] as this questionnaire has a well predictive power [20, 21]. The likelihood of having moderate or severe OSA increases with each point increase in the STOP-bang score. In our hospital we observed that patients not using CPAP due to the absence of a diagnosis for OSA based on a PSG test had more complications and increased length of hospital stay (LOS) than those who did. An increased LOS limits the capacity of the hospital system and hence contributes to a poorer economic performance. In this study we hypothesize that morbidly obese patients diagnosed with OSA by a PSG test and using CPAP have fewer perioperative complications and a shorter hospital stay than those who have a medical history which meets at least three STOP-Bang criteria and who are not using CPAP.

## Patients and methods

After Institutional Review Board (IRB) approval for the study was obtained, data on all patients undergoing bariatric surgery between January 2009 and November 2013 were collected. The need for consent was waived by our IRB due to the entirely retrospective nature of the study. During the study period, based on a prospective database, 693 patients underwent bariatric surgery in the Department of General, Endocrine and Transplant Surgery, Medical University of Gdańsk, Poland. The scheduling of patients for the operation was based on their arrival in the hospital, on a first-come first-served basis. Criteria for patient eligibility for bariatric surgery include the following [22]: (1) age range 18–65; (2) weight of >45 kg above the ideal body weight for sex and height; (3) body mass index (BMI) of >40 kg/m^2^ by itself or >35 kg/m^2^ in the case of an associated comorbidity such as diabetes or sleep apnea; (4) obesity-related health problems; (4) reasonable attempts at other weight loss techniques; (5) no psychiatric or drug dependency problems; (6) capacity to understand the risks and commitment associated with the surgery; (7) pregnancy not anticipated in the first 2 years following surgery. Criteria for exclusion from the study were emergency or urgent surgery required within 48 h and patients with trauma or who were critically ill patients. The performed surgical procedures were Roux-en-Y gastric bypass (RYGB) and laparoscopic sleeve gastrectomy (LSG). As a part of the bariatric protocol, all patients, both those diagnosed and not diagnosed for OSA, were asked to fill in the STOP-Bang questionnaire. STOP-Bang scoring was conducted by trained personnel.

### Patient groups

We divided the patients into three groups, as follows:

Group A: This group consisted of patients with preoperatively diagnosed OSA based on PSG test results. The test was performed in our hospital or in other clinics before these patients allowed to enrol in the bariatric pathway program, and the results were added to the patient file. The patients in this group used CPAP at home pre- and postoperatively, and none of these patients had been treated surgically to solve OSA. These patients also brought their own CPAP mask to the hospital. Patients accepted for surgery were informed by the surgeon about the health benefits of using CPAP, and they were asked to immediately inform the outpatient department when they were not able to comply. This (and the reason why) was noted in the patient file. The CPAP apparatus records compliance data in order to provide the necessary information, such as usage (in hours) per night. Current trends define compliance as 4 h per night as an average over all nights observed [23]. We measured compliance from the second week of CPAP usage until a day before the scheduled surgery.
Group B: This group consists of patients not diagnosed for OSA by use of the PSG test and patients who underwent a PSG test and were not diagnosed for OSA. Patients in group B complied to at least three STOP-Bang criteria and did not use CPAP.
Group C: This group consists of patients scoring one to two items on the STOP-Bang questionnaire, indicating that they have a low risk for OSA.

In all groups during the preoperative, intraoperative and postoperative phases, standardized multimodal analgesia, antiemesis and thromboprophylaxis were given. For induction, this included piritramide 20 mg, propofol 200–400 mg, suxamethonium 100–200 mg, granisetron 3 mg, dexamethasone 8 mg. For maintenance, this included remifentanyl and desflurane. Postoperatively, all patients were stimulated for early mobilization. Group A patients with proven OSA had their own CPAP mask with them; the mask was applied immediately after extubation in the operating room or approximately after 30 min in the post-anesthesia care unit (PACU) for a minimum of 8 h. During the night the CPAP mask was also used. After surgery, these patients were monitored using pulse oximetry in the PACU and in the surgical ward.

### Study endpoints

For the three groups, we analyzed the following data on the day of surgery and during postoperative hospital stay:

1. Pulse oximetry (SpO_2_; blood oxygen saturation): postoperative period until the morning of the second day after surgery (days 0–2, 08:00 to 09:00 AM);
2. Respiratory rate: postoperative period until the first morning after surgery (08:00 to 10:00 AM);
3. LOS: day of admission until day of discharge;
4. Patient outcome variables: acute myocardial infarction, cerebrovascular accident, pneumonia, reintubations, ICU admission rates.

We used the following definitions for the different outcome variables. Pneumonia was diagnosed by clinical features (e.g., cough, fever, pleuritic chest pain) and by lung imaging (X-ray). Myocardial Infarction was diagnosed when there is a rise and/or fall of cardiac biomarkers, along with supportive evidence in the form of typical symptoms, suggestive electrocardiographic changes, or imaging evidence of new loss of viable myocardium or new regional wall motion abnormality. Symptoms are chest pain and dyspnea [23]. Stroke was defined as rapidly developing clinical signs of focal (or global) disturbance(s) in cerebral function lasting more than 24 h (except in cases of sudden death or if the development of symptoms is interrupted by a surgical intervention), with no apparent cause other than a vascular origin: it includes patients presenting clinical signs and symptoms suggestive of subarachnoid hemorrhage, intracerebral hemorrhage, or cerebral ischemic necrosis [24]. Hypertension was defined as blood pressure at or above 140/90 mmHg. Reasons for reintubation were, for example, hypoxia, hypercapnia, and exhaustion; reasons for ICU admissions were, for example, hypotension, respiratory problems, (abdominal) sepsis, bleeding, and re-intervention. Surgical criteria for hospital discharge included normal mobility, normal respiratory function, normal cardiac function, no signs of infection, low pain score (e.g. visual analog score), normal bowel function, and absence of nausea.

SpO_2_ was monitored continuously (also during asleep) postoperatively until the morning of the second night after surgery (08:00–10:00 AM), and measurements were logged every 5 min. The SpO_2_ levels presented in the tables were measured without oxygen administration to avoid possible confounding factors. Consequently, if the saturation level demanded the temporary administration of oxygen, the SpO_2_ levels during this period were excluded from the calculations. The number of patients who needed oxygen and the number of times after surgery the oxygen was required were counted and presented in the results. The respiratory rate (breaths per minute) were measured (during sleep and wakefulness) using electrocardiography-derived respiration during the immediate postoperative period until the first morning after surgery (08:00–10:00 AM).

Follow-up was verified and adverse events reviewed, including death, unanticipated transfer or admission to hospital within 30 days after surgery.

### Statistics

Statistical analysis was performed using the SPSS Statistics ver. 20 program (SPSS Inc., Chicago, IL). One-way analysis of variance was performed to determine the significance of means among the groups. After testing for homogeneity of variance, we used Tukey’s HSD post hoc test odds-ratio test to accept or reject the null hypothesis that portions are equal. *p* values of <0.05 were considered to be statistically significant.

## Results

A total of 693 patients underwent bariatric surgery (356 RYGB, 337 patients LSG). All patients underwent bariatric operations without any surgical complications, and none of the patients were unexpectedly transferred or admitted to our or another hospital within 30 days after surgery. Table 1 presents the patient characteristics and the preoperative existing comorbidities. Patients in groups A and B did not differ significantly in terms of demographics and the presence of comorbidities (*p* > 0.05). The demographics and presence of comorbidities did differ between group C and group A/B for hypertension only [*p* = 0.042 (groups A–C) vs. *p* = 0.008 (groups B–C)].

**Table 1:** Patient characteristics

Patient characteristics	Group A (*n* = 99)	Group B (*n* = 182)	Group C (*n* = 412)
Age (years)^a^	44.5 (7.0)	46.6 (5.3)	46.0 (5.8)
Sex: male/female (*n*, %)	72/27 (72/28.0)	129/53 (70.9/29.1)	285/127 (69.2/30.8)
Body mass index (kg/m^2^)^a^	42.6 (2.6)	43.5 (2.1)	43.2 (3.1)
Comorbidities (*n*, %)			
Chronic obstructive pulmonary disease	7 (7.9	14 (7.6)	18 (4.4)
Asthma	9 (10.1)	16 (8.9)	21 (5.1)
Diabetes mellitus type 2	14 (15.7)	31 (17.0)	65 (15.8)
Coronary artery disease	15 (16.9)	25 (13.7)	49 (11.9)
Hypertension	31 (34.8)	58 (31.9)	89 (21.6)

^a^Values given as the mean ± standard deviation (SD)

Table 2 shows the STOP-BANG results per group differentiated to the level of risk for OSA. The odd-ratio shows no significant difference between patients in the different risk levels [*p* = 0.1137 (moderate risk group A–B) vs. *p* = 0.4687 (high risk group A–B).

**Table 2:** Distribution of STOP-BANG scores

Group A	Group B	Group C
Moderate risk: 49 (49.4 %) High risk: 41 (41.4 %)	Moderate risk: 112 (61.5 %) High risk: 70 (38.5 %)	Low risk: 412 (100 %)

Table 3 presents the distribution of all the STOP-Bang factors for each patient group, Table 4 shows the compliance rate versus the apnea/hypopnea index (AHI) per group, Table 5 shows the data on SpO_2_ and respiratory rate, and in Table 6 we present other relevant characteristics of the groups. There were no patients with acute myocardial infarction or cerebrovascular accident.

**Table 3:** Distribution of positive scored STOP-Bang factors for each patient group

STOP-Bang factors	Group A	Group B	Group C
**S**noring? Do you snore loudly (loud enough to be heard through closed doors or your partner has to wear ear plugs or elbow you at night)?	91 (91.9 %)	163 (89.0 %)	46 (11.2 %)
**T**ired? Do you often feel tired, fatigued, or sleepy during the daytime?	95 (95.9 %)	145 (80.0 %)	28 (6.8 %)
**O**bserved ? Has anyone observed you stop breathing during your sleep?	71 (71.7 %)	131 (72.0 %)	34 (8.3 %)
**P**ressure ? Do you have or are being treated for high blood pressure?	86 (86.9 %)	171 (94.0 %)	22 (5.3 %)
**B**ody mass index of >35 kg/m^2^?	97 (98.0 %)	182 (100 %)	412 (100 %)
**A**ge older than 50 years?	21 (10.1 %)	44 (24.2 %)	69 (16.7 %)
**N**eck size large? For male, is your shirt collar 17 inches or larger? For female, is your shirt collar 16 inches or larger?	94 (94.9 %)	103 (97.8 %)	39 (9.5 %)
**G**ender = male?	72 (72.7 %)	129 (70.9 %)	285 (69.2 %)

Based on STOP-Bang questionnaire [19, 20]. All data are presented as the score with the percentage in parenthesis

**Table 4:** Patients in group A: compliance versus the apnea/hypopnea index

AHI index	No. of patients who complied	No. of patients who did not comply	Total
AHI >30	49	2	51
AHI >15	29	3	32
AHI 5–15	4	12	16
Total	81	17	99

*AHI* Apnea/hypopnea index

**Table 5:** Mean blood oxygen saturation and respiratory rates in the patient groups

SpO_2_ level/RR	Group A (*n* = 99)	Group B (*n* = 182)	Group C (*n* = 412)	Differences group A–B (*p* values)	Differences group B–C (*p* values)
SpO_2_ (%) day 0	92.1 (1.4)	90.8 (1.6)	94.8 (1.2)	<0.0001*	<0.0001*
SpO_2_ (%) day 1	94.3 (1.2)	90.6 (1.3)	96.6 (1.3)	<0.0001*	<0.0001*
SpO_2_ (%) day 2	96.6 (1.1)	94.8 (1.4)	98.4 (1.5)	<0.0001*	<0.0001*
RR (breaths/min) on day 0	15.8 (1.9)	12.4 (2.4)	16.1 (0.9)	<0.0001*	<0.0001*
RR (breaths/min) day 1	16.6 (1.4)	13.6 (2.9)	18.0 (0.7)	<0.0001*	<0.0001*

Data are given as the mean with the SD in parenthesis

*SpO*_2_ Blood oxygen saturation,* RR* respiration risk

* Significant at the 5 % level

**Table 6:** Characteristics of the groups

Characteristics	Group A (*n* = 99)	Group B (*n* = 182)	Group C (*n* = 412)	Differences Group A–B (*p* values)	Differences Group B–C (*p* values)
Hypertension (*n*)	11 7 AHI > 30 4 AHI > 15	21 8 moderate risk 12 high risk	12	0.9142	<0.0001*
Deaths (*n*)	0	2	0	0.5142	0.3807
Length of stay (mean, SD)	3.2 (0.7)	4.1 (0.5)	2.5 (0.6)	0.0001*	<0.0001*
Pneumonia (*n*)	2 2 AHI > 30	17 6 moderate risk 11 high risk	4	0.040*	<0.0001*
Reintubations (*n*)	0	7 7 high risk	0	0.1442	0.0098*
ICU admissions (n)	0	2 2 high risk	0	0.5142	0.0873*

* Significant at the 5 % level

### Group A

A total of 122 patients were originally included in group A. However, a search of the patient records revealed that 23 patients had reported that they did not use CPAP at all, or only some nights per week (varying from 0–4 nights per week) for 0–2 h per night. These patients were excluded from this group and included in group B, leaving 99 patients (57 RYGB, 42 LSG) in group A.

The compliance rate of this group was 81.8 % {81 patients, average usage 5.9 [standard deviation (SD) 0.8] h/night; minimum–maximum usage 4.0–9.1 h}. Based on the PSG test we found that 51 patients had severe sleep apnea (AHI >30), 32 patients had moderate sleep apnea (AHI >15), and 16 had mild sleep apnea (AHI 5–15).

In all cases, patients could be safely discharged from intensive monitoring (reaching the SpO_2_ >90 %) on the second day after the bariatric operation. Only in one case, did a respiratory tract complication (pulmonary embolism) occur; this patient was treated accordingly. No other patient was administered oxygen during the postoperative period. The mean length of LOS in group A for RYGB and LSG was 3.2 (0.7) and 3.1 (0.4) days, respectively.

### Group B

A total of 182 patients were included in group B, of whom 112 (53 RYGB, 49 LSG) scored three to four items on the STOP-Bang questionnaire (moderate risk) and 70 (30 RYGB, 40 LSG) scored five to eight items (high risk). Included in these 182 patients were 23 patients originally from group A who reported that they did not use CPAP (at all).

In this group the mean value of SpO_2_ on days 0–2 postoperatively was significantly lower than that of group A patients (*p* < 0.0001). On days 0–1 the respiratory rate in group B had a larger variation and was significantly lower than those of group A and C. Sixteen patients received oxygen in the postoperative period; this occurred between 1 and 14 h after surgery and the duration of oxygen administration varied between 20 and 45 min. Further analysis (not shown) revealed that there was no difference between the RGB/LSG patients. The mean LOS was 4.1 (0.5)/4.2 (0.6) days (RGB/LSG), which is significantly (*p* < 0.001) longer than that for group A. The percentage of complications did not differ significantly from that of the other groups. In this group we also noted two cases of sudden death, both of which took place during the night on the second postoperative day. These patients (I and II) were two males aged 34 and 41 years, respectively. Patients (I/II) had a BMI of 44/46 kg/m^2^ and had scored four/six items on the STOP-Bang. Both had hypertension and diabetes and snored loudly during hospitalization (before and after surgery). Both patients also presented with signs and symptoms of dyspnea and hypoxia (SpO_2_ < 90 %) on the day of surgery (day 0) and the first postoperative day. For patient I, we also observed high blood pressure which required antihypertensive drugs. Autopsy revealed that both patients died because of sudden cardiac death caused by asphyxia.

### Group C

There were 412 (221 RYGB, 181 LSG) patients in this group, all of whom scored one or two items on the STOP-Bang. In this group the respiratory parameters and LOS were significantly better than in those group B, and there was no significant difference with group A.

## Discussion

Our results show that morbidly obese patients diagnosed with OSA by a PSG test and who are using CPAP have fewer perioperative complications and a shorter hospital stay than patients whose medical history meets at least three STOP-Bang criteria and who are not using CPAP. They also indicate that when the severity of OSA is known one can take more appropriate measures to reduce the risk of complications and reduce LOS in hospital. Our findings confirm that it is essential to address the burden of morbidly obese patients diagnosed with OSA to public healthcare systems and to develop cost-effective case-finding strategies and feasible treatments for OSA [1]. Our findings are opposite to those reported by Kaw et al. [25] who reported that non-cardiac patients with OSA (AHI ≥ 5) were are at higher risk of postoperative hypoxemia, ICU transfers, and longer LOS. However, these authors did not differentiate between the specific type of procedure (bariatric), and the average BMI of these patients was lower. It is known that the more specific the group of patients, the more precisely the outcomes can be defined. Hence, this may explain the difference in complications and LOS between the two studies. In our study we found that the higher the AHI index, the higher the compliance. Earlier findings are not clear in terms of determining whether there is a causal relation between AHI and compliance [33]. Our results confirm those of earlier studies demonstrating a relation between diagnosed OSA (not using CPAP) and an increased incidence of postoperative complications, with the most frequent being oxygen desaturation [27–29].

In our study we show a negative effect on LOS whereas other studies [29] showed a mixed impact. For the final diagnosis of OSA, PSG tests are essential. To our knowledge, there have as yet been no double-blinded randomized studies in a preoperative assessment setting showing the outcomes of PSG tests versus questionnaires. It would appear reasonable to ask if a PSG is always necessary when a patient scores three or more items on the STOP-Bang questionnaire. The PSG test is relatively costly [30], and the STOP-Bang does has highly prognostic value for OSA [20, 21]. From the economic point of view the use of CPAP also reduces the LOS in hospital and hence reduces costs per patients admitted. All other things being equal, a shorter stay may reduce the cost per discharge and shift care from inpatient to less expensive post-acute settings. Also, OSA is related to severe respiratory complications and ICU admission. Reducing costs by identifying and treating risk patients with CPAP and consequently avoiding the expensive use of ICU facilities is desirable [31, 32].

There are a number of limitations to our study. As this is a retrospective study, it can show association but not causality. Moreover, the study population is relatively small. Larger randomized case–control studies are needed to confirm our results. Although we have information about the STOP-Bang results for groups A and B, the severity for OSA in Group A is objectively determined by the PSG test. Although we may assume that patients in Group B scoring five to eight items on the STOP-Bang have an elevated risk for OSA, the severity of OSA is still not clear. It may be that these patients on average have a higher median AHI score when diagnosed for OSA and that this result may then (partially) explain the higher postoperative complications in group B.

Based on our results, we conclude that patients meeting at least three STOP-BANG criteria can be expected to have higher postoperative complications and an increased LOS than patients using CPAP.

## References

[1] Young T, Peppard PE, Gottlieb DJ (2002). Epidemiology of obstructive sleep apnea: A population health perspective. Am J Respir Crit Care Med.

[2] Frey WC, Pilcher J (2003). Obstructive sleep-related breathing disorders in patients evaluated for bariatric surgery. Obes Surg.

[3] Schwab RJ, Pasirstein M, Pierson R, Mackley A, Hachadoorian R, Arens R, Maislin G, Pack AI (2003). Identification of upper airway anatomic risk factors for obstructive sleep apnea 375 with volumetric magnetic resonance imaging. Am J Respir Crit Care Med.

[4] Gami AS, Howard DE, Olson EJ, Somers VK (2005). Day-night pattern of sudden death in obstructive sleep apnea. N Engl J Med.

[5] Duran-Cantolla J, Aizpuru F, Martinez-Null C, Barbe-Illa F (2009). Obstructive sleep apnea/hypopnea and systemic hypertension. Sleep Med Rev.

[6] Gami AS, Olson EJ, Shen WK, Wright RS, Ballman KV, Hodge DO, Herges RM, Howard DE, Somers VK (2013). Obstructive Sleep Apnea and the risk of sudden cardiac death A Longitudinal study of 10,701 adults. J Am Coll Cardiol..

[7] Kaw R, Chung F, Pasupuleti V, Mehta J, Gay PC (2012). Hernandez AV meta-analysis of the association between obstructive sleep apnoea and postoperative outcome. Br J Anaesth.

[8] Weingarten TN, Flores AS, McKenzie JA, Nguyen LT, Robinson WB, Kinney TM, Siems BT, Wenzel PJ, Sarr MG, Marienau MS, Schroeder DR, Olson EJ, Morgenthaler TI, Warner DO, Sprung J (2011). Obstructive sleep apnoea and perioperative complications in bariatric patients. Br J Anaesth.

[9] Tuomilehto H, Seppä J, Uusitupa M (2013). Obesity and obstructive sleep apnea—clinical significance of weight loss. Sleep Med Rev.

[10] Paje DT, Kremer MJ (2006). The perioperative implications of obstructive sleep apnea. Orthop Nurs.

[11] Lopes Neto JM, Brandão LO, Loli A, Leite CV, Weber SA (2013). Evaluation of obstructive sleep apnea in obese patients scheduled for bariatric surgery. Acta Cir Bras.

[12] Khan A, King WC, Patterson EJ, Laut J, Raum W, Courcoulas AP, Atwood C, Wolfe BM (2013). Assessment of obstructive sleep apnea in adults undergoing bariatric surgery in the longitudinal assessment of bariatric surgery-2 (LABS-2) study. J Clin Sleep Med.

[13] Hallowell PT, Stellato TA, Schuster M, Graf K, Robinson A, Crouse C, Jasper JJ (2007). Potentially life-threatening sleep apnea is unrecognized without aggressive evaluation. Am J Surg.

[14] Palla A, Digiorgio M, Carpenè N, Rossi G, D’Amico I, Santini F, Pinchera A (2009). Sleep apnea in morbidly obese patients: prevalence and clinical predictivity. Respiration.

[15] Epstein LJ, Kristo D, Strollo PJ, Friedman N, Malhotra A, Patil SP, Ramar K, Rogers R, Schwab RJ, Weaver EM, Weinstein MD (2009). Clinical guideline for the evaluation, management and long-term care of obstructive sleep apnea in adults. J Clin Sleep Med.

[16] O’Keeffe T, Patterson EJ (2004). Evidence supporting routine polysomnography before bariatric surgery. Obes Surg.

[17] Shiota S, Inoue Y, Takekawa H, Kotajima M, Nakajyo M, Usui C, Yoshioka Y, Koga T, Takahashi K (2014). Effect of continuous positive airway pressure on regional cerebral blood flow during wakefulness in obstructive sleep apnea. Sleep Breath.

[18] Bakker JP, Balachandran JS, Tecilazich F, Deyoung PN, Smales E, Veves A, Malhotra A (2013). Pilot study of the effects of bariatric surgery and continuous positive airway pressure treatment on vascular function in obese subjects with obstructive sleep apnoea. Intern Med J.

[19] Chung F, Yegneswaran B, Liao P, Chung SA, Vairavanathan S, Islam S, Khajehdehi A, Shapiro CM (2008). STOP questionnaire: a tool to screen patients for obstructive sleep apnea. Anesthesiology.

[20] Chung F, Yang Y, Liao P (2013). Predictive performance of the STOP-Bang score for identifying obstructive sleep apnea in obese patients. Obes Surg..

[21] van Zeller M, Severo M, Santos AC, Drummond M. 5-Years APAP adherence in OSA patients—Do first impressions matter? Respir Med. 2013.107(12):2046–52. 10.1016/j.rmed.2013.10.011 24169074

[22] http://www.ifso.com. Assessed on 7 Apr 2014.

[23] www.uptodate.com/contents/criteria-for-the-diagnosis-of-acute-myocardial-infarction

[24] www.cuore.iss.it/eurociss/reg_ictus/pdf/ictus_criteri-diagnostici.pdf

[25] Weaver TE, Maislin G, Dinges DF, Bloxham T, George CF, Greenberg H, Kader G, Mahowald M, Younger J, Pack AI (2007). Relationship between hours of CPAP use and achieving normal levels of sleepiness and daily functioning. Sleep.

[26] Kaw R, Pasupuleti V, Walker E, Ramaswamy A, Foldvary-Schafer N (2012). Postoperative complications in patients with obstructive sleep apnea. Chest..

[27] Memtsoudis SG, Liu SS, Ma Y, Chiu YL, Walz JM, Gaber-Baylis LK, Mazumdar M (2011). Perioperative pulmonary outcomes in patients with sleep apnea after noncardiac surgery. Anesth Analg.

[28] Liao P, Yegneswaran B, Vairavanathan S, Zilberman P, Chung F (2009). Postoperative complications in patients with obstructive sleep apnea: a retrospective matched cohort study. Can J Anesth.

[29] Mokhlesi B, Hovda MD, Vekhter B, Arora VM, Chung F, Meltzer DO (2013). Sleep-disordered breathing and postoperative outcomes after bariatric surgery: Analysis of the nationwide inpatient sample. Obes Surg.

[30] Deutsch PA, Simmons MS, Wallace JM (2006). Cost-effectiveness of split-night polysomnography and home studies in the evaluation of obstructive sleep apnea syndrome. J Clin Sleep Med.

[31] de Quelerij M, van Velzen C, Luitwieler R, Meijer N, Gadiot RPM, van der Voet J, van der Klooster J, de Feiter PW, Mannaerts G, Verbrugge SJC (2012). Prevalence and determinants of complications in a bariatric ICU population. NTVA.

[32] Van den Broek RJ, Buise MP, van Dielen FM, Bindels AJ, van Zundert AA, Smulders JF (2009). Characteristics and outcome of patients admitted to the ICU following bariatric surgery. Obes Surg.

[33] Yetkin O, Kunter E, Gunen H (2008). CPAP compliance in patients with obstructive sleep apnea syndrome. Sleep Breath.

